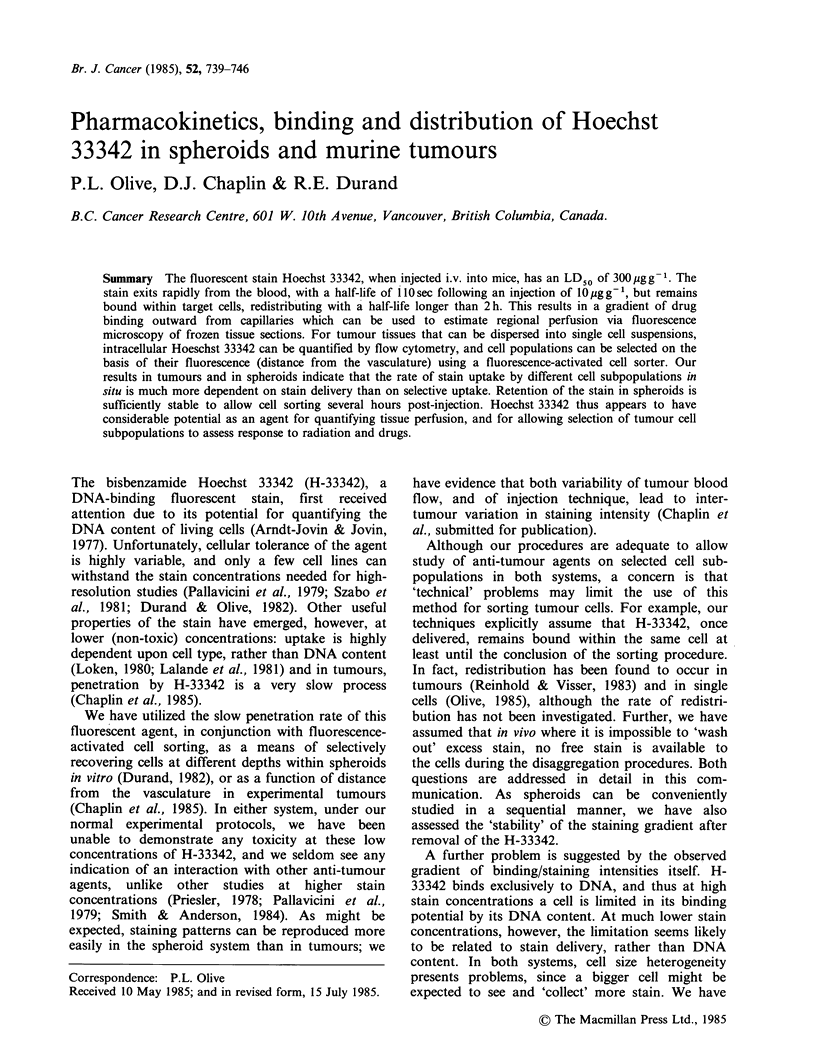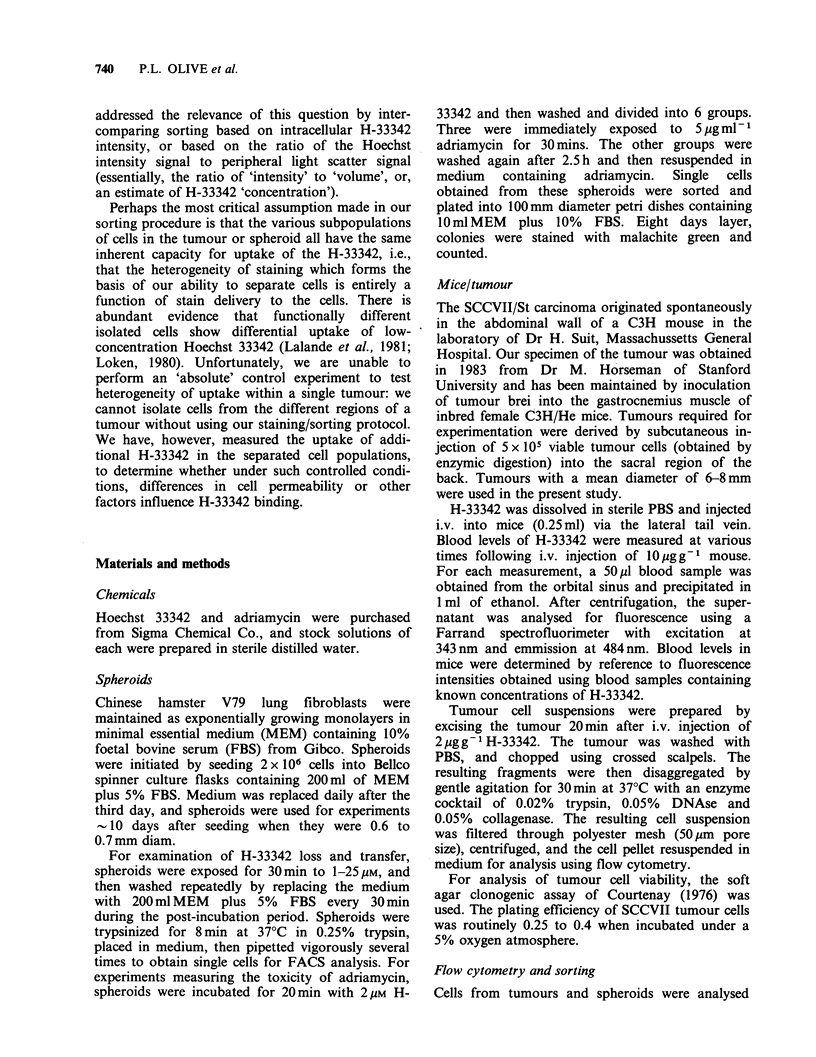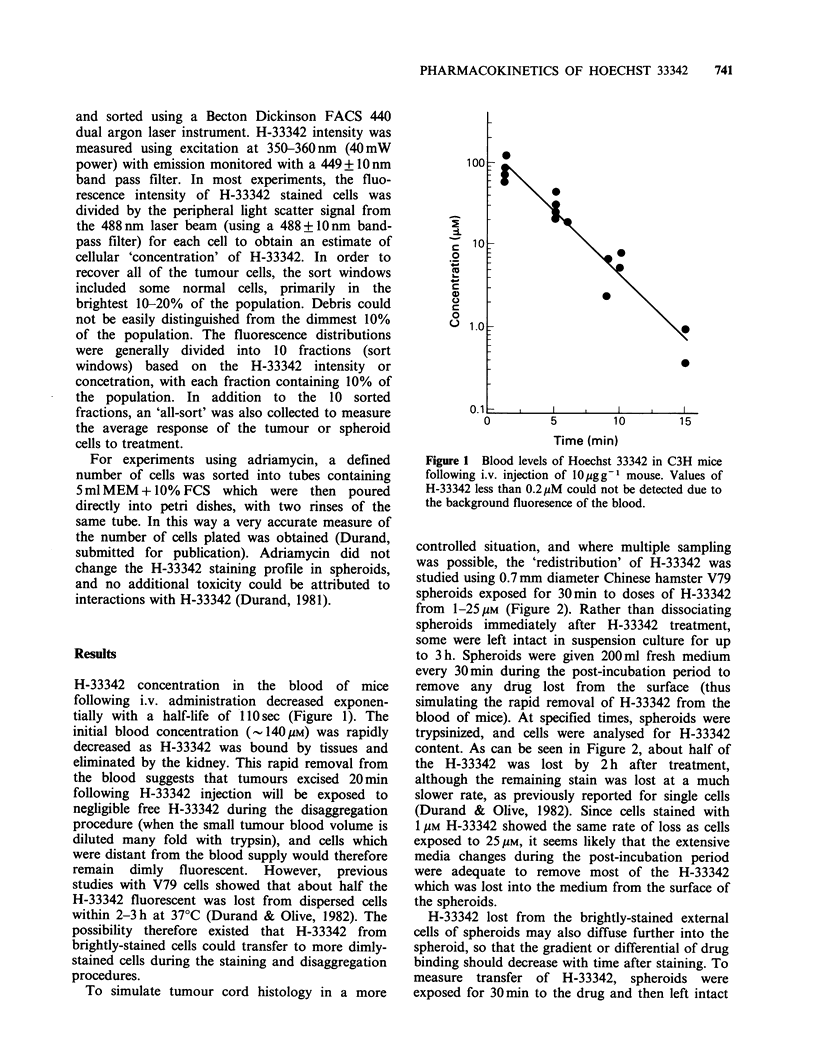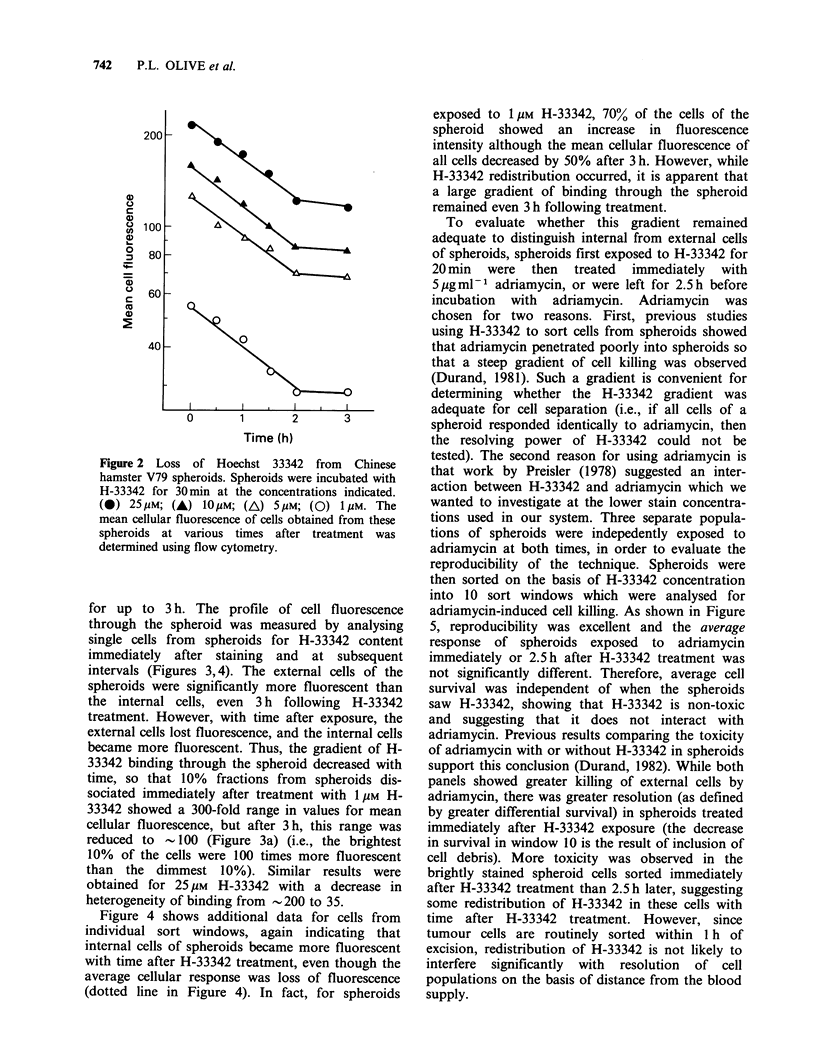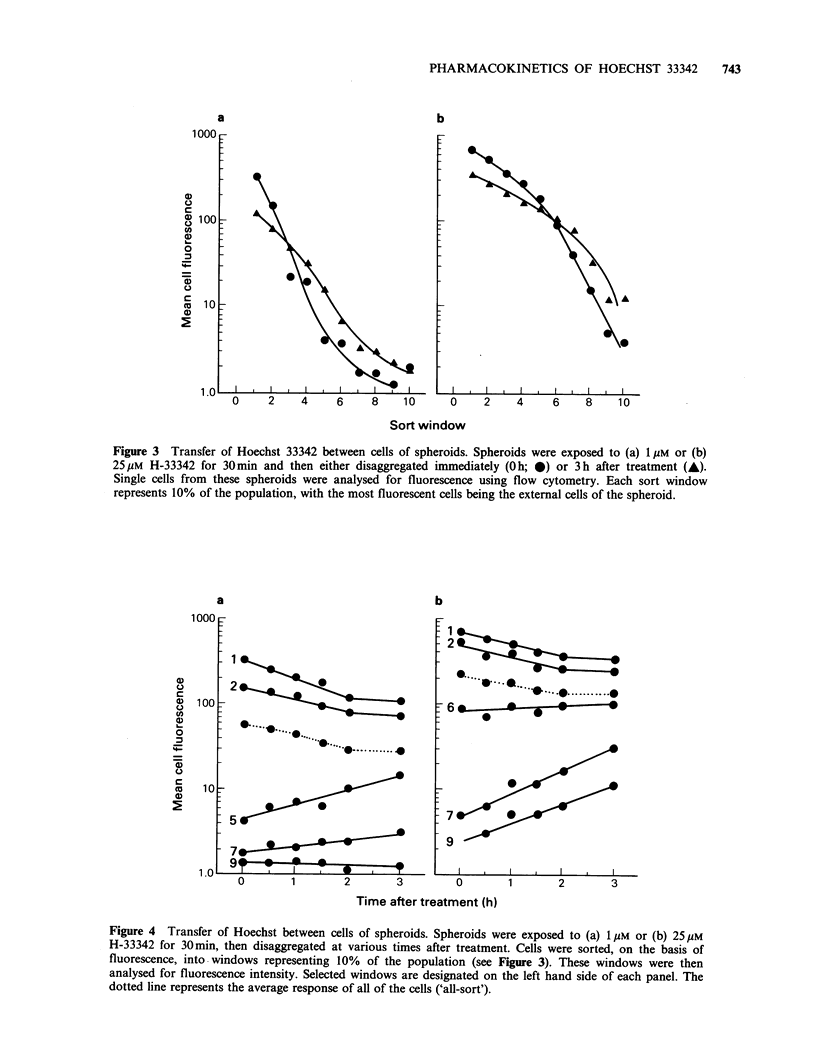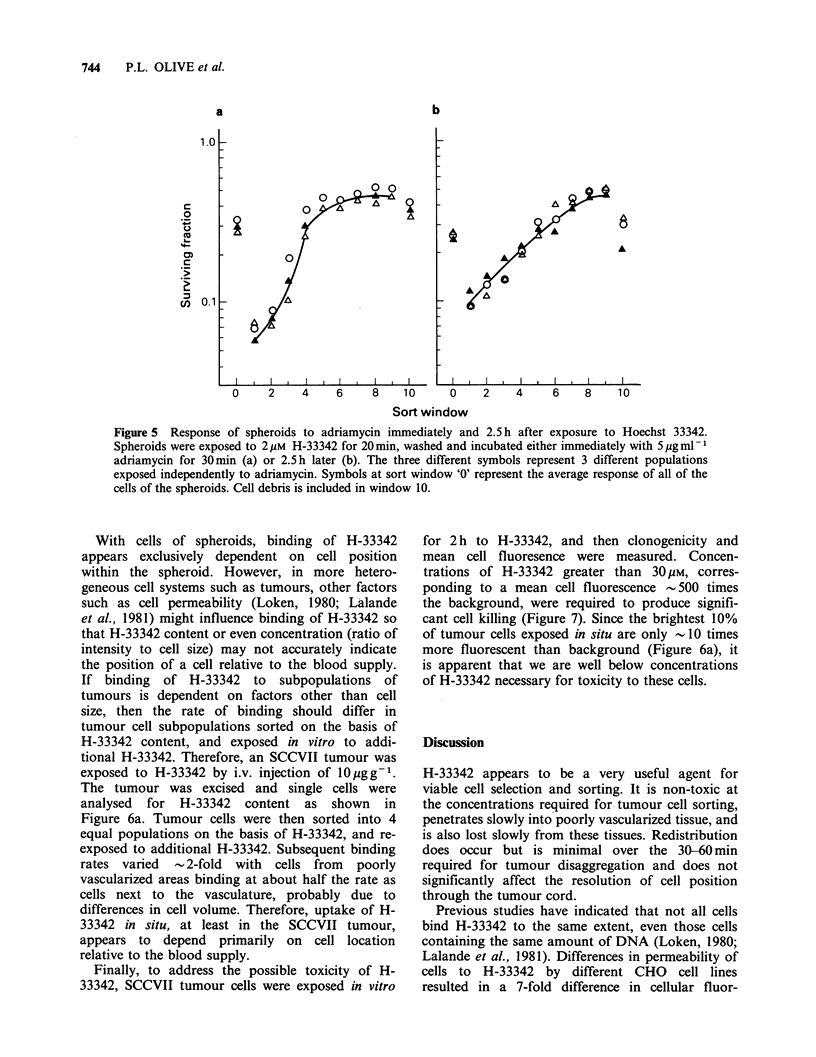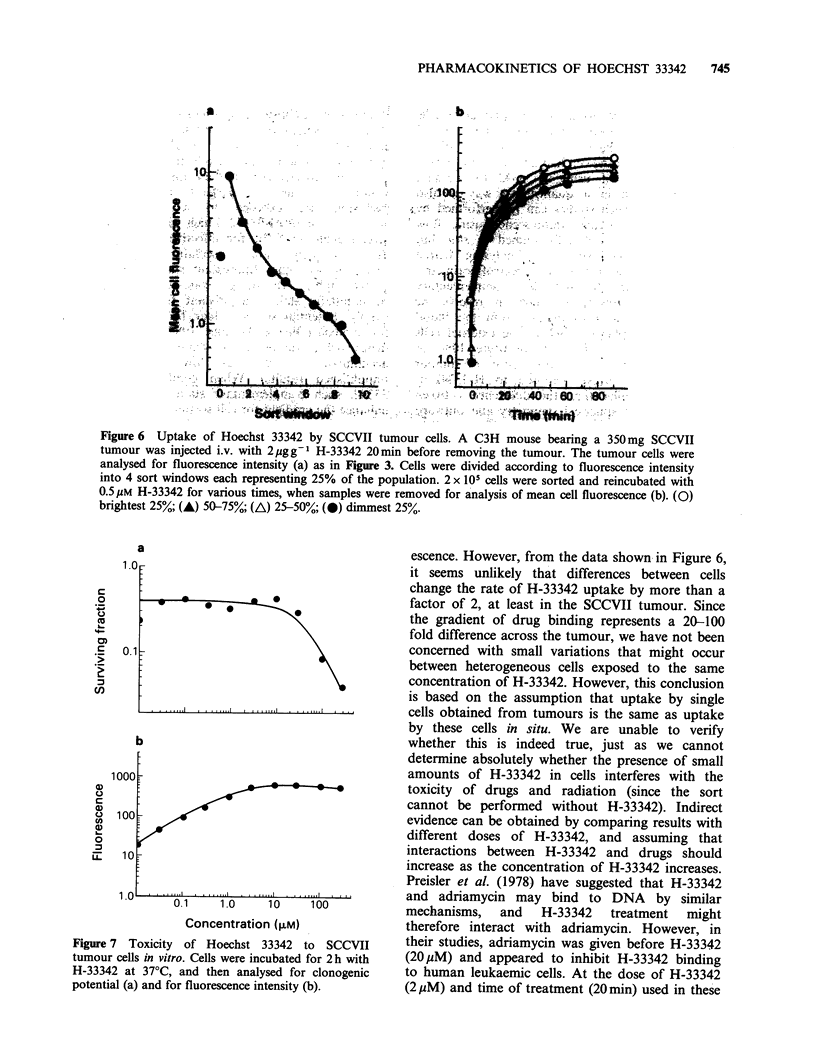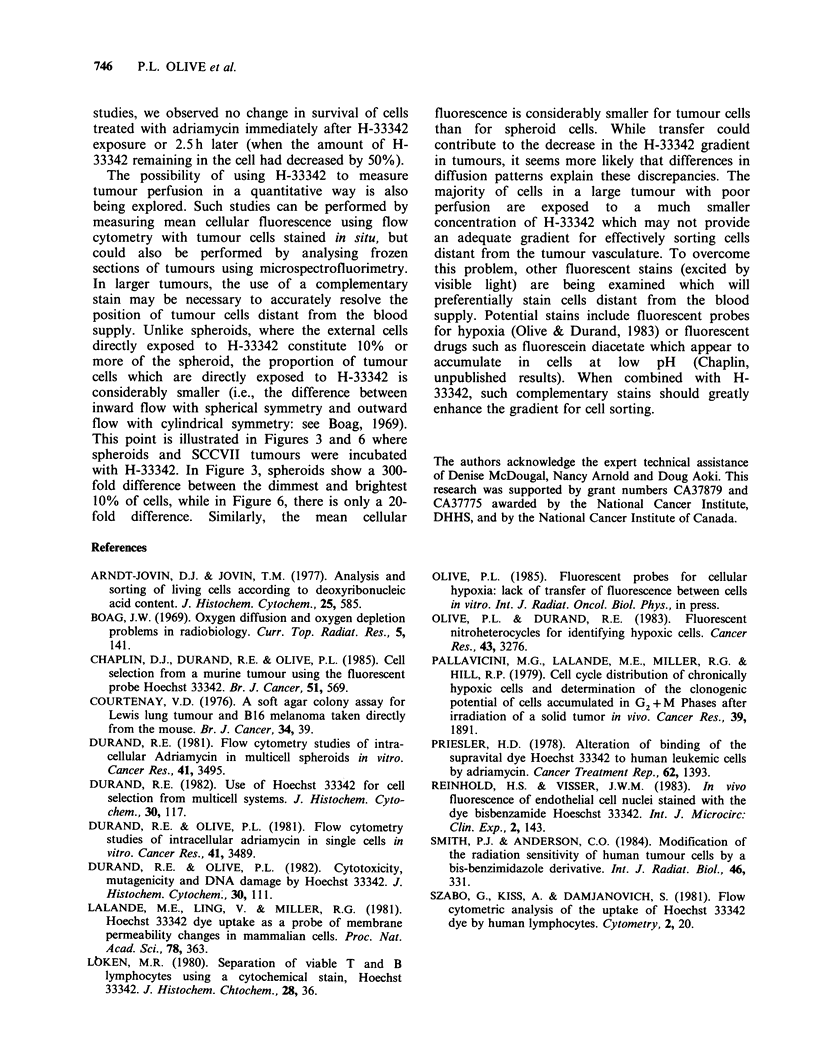# Pharmacokinetics, binding and distribution of Hoechst 33342 in spheroids and murine tumours.

**DOI:** 10.1038/bjc.1985.252

**Published:** 1985-11

**Authors:** P. L. Olive, D. J. Chaplin, R. E. Durand

## Abstract

The fluorescent stain Hoechst 33342, when injected i.v. into mice, has an LD50 of 300 micrograms g-1. The stain exits rapidly from the blood, with a half-life of 110 sec following an injection of 10 micrograms g-1, but remains bound within target cells, redistributing with a half-life longer than 2 h. This results in a gradient of drug binding outward from capillaries which can be used to estimate regional perfusion via fluorescence microscopy of frozen tissue sections. For tumour tissues that can be dispersed into single cell suspensions, intracellular Hoeschst 33342 can be quantified by flow cytometry, and cell populations can be selected on the basis of their fluorescence (distance from the vasculature) using a fluorescence-activated cell sorter. Our results in tumours and in spheroids indicate that the rate of stain uptake by different cell subpopulations in situ is much more dependent on stain delivery than on selective uptake. Retention of the stain in spheroids is sufficiently stable to allow cell sorting several hours post-injection. Hoechst 33342 thus appears to have considerable potential as an agent for quantifying tissue perfusion, and for allowing selection of tumour cell subpopulations to assess response to radiation and drugs.


*Br. J. Cancer* (1985), **52**, 739–746

### Pharmacokinetics, binding and distribution of Hoechst 33342 in spheroids and murine tumours

P.L. Olive, D.J. Chaplin & R.E. Durand

*B.C. Cancer Research Centre, 601 W. 10th Avenue, Vancouver, British Columbia, Canada.*

**Summary** The fluorescent stain Hoechst 33342, when injected i.v. into mice, has an $LD_{50}$ of $300\,\mu g\,g^{-1}$. The stain exits rapidly from the blood, with a half-life of 110 sec following an injection of $10\,\mu g\,g^{-1}$, but remains bound within target cells, redistributing with a half-life longer than 2 h. This results in a gradient of drug binding outward from capillaries which can be used to estimate regional perfusion via fluorescence microscopy of frozen tissue sections. For tumour tissues that can be dispersed into single cell suspensions, intracellular Hoeschst 33342 can be quantified by flow cytometry, and cell populations can be selected on the basis of their fluorescence (distance from the vasculature) using a fluorescence-activated cell sorter. Our results in tumours and in spheroids indicate that the rate of stain uptake by different cell subpopulations *in situ* is much more dependent on stain delivery than on selective uptake. Retention of the stain in spheroids is sufficiently stable to allow cell sorting several hours post-injection. Hoechst 33342 thus appears to have considerable potential as an agent for quantifying tissue perfusion, and for allowing selection of tumour cell subpopulations to assess response to radiation and drugs.

The bisbenzamide Hoechst 33342 (H-33342), a DNA-binding fluorescent stain, first received attention due to its potential for quantifying the DNA content of living cells (Arndt-Jovin & Jovin, 1977). Unfortunately, cellular tolerance of the agent is highly variable, and only a few cell lines can withstand the stain concentrations needed for high-resolution studies (Pallavicini *et al.*, 1979; Szabo *et al.*, 1981; Durand & Olive, 1982). Other useful properties of the stain have emerged, however, at lower (non-toxic) concentrations: uptake is highly dependent upon cell type, rather than DNA content (Loken, 1980; Lalande *et al.*, 1981) and in tumours, penetration by H-33342 is a very slow process (Chaplin *et al.*, 1985).

We have utilized the slow penetration rate of this fluorescent agent, in conjunction with fluorescence-activated cell sorting, as a means of selectively recovering cells at different depths within spheroids *in vitro* (Durand, 1982), or as a function of distance from the vasculature in experimental tumours (Chaplin *et al.*, 1985). In either system, under our normal experimental protocols, we have been unable to demonstrate any toxicity at these low concentrations of H-33342, and we seldom see any indication of an interaction with other anti-tumour agents, unlike other studies at higher stain concentrations (Priesler, 1978; Pallavicini *et al.*, 1979; Smith & Anderson, 1984). As might be expected, staining patterns can be reproduced more easily in the spheroid system than in tumours; we have evidence that both variability of tumour blood flow, and of injection technique, lead to inter-tumour variation in staining intensity (Chaplin *et al.*, submitted for publication).

Although our procedures are adequate to allow study of anti-tumour agents on selected cell sub-populations in both systems, a concern is that 'technical' problems may limit the use of this method for sorting tumour cells. For example, our techniques explicitly assume that H-33342, once delivered, remains bound within the same cell at least until the conclusion of the sorting procedure. In fact, redistribution has been found to occur in tumours (Reinhold & Visser, 1983) and in single cells (Olive, 1985), although the rate of redistribution has not been investigated. Further, we have assumed that *in vivo* where it is impossible to 'wash out' excess stain, no free stain is available to the cells during the disaggregation procedures. Both questions are addressed in detail in this communication. As spheroids can be conveniently studied in a sequential manner, we have also assessed the 'stability' of the staining gradient after removal of the H-33342.

A further problem is suggested by the observed gradient of binding/staining intensities itself. H-33342 binds exclusively to DNA, and thus at high stain concentrations a cell is limited in its binding potential by its DNA content. At much lower stain concentrations, however, the limitation seems likely to be related to stain delivery, rather than DNA content. In both systems, cell size heterogeneity presents problems, since a bigger cell might be expected to see and 'collect' more stain. We have

Correspondence: P.L. Olive
Received 10 May 1985; and in revised form, 15 July 1985.

© The Macmillan Press Ltd., 1985

addressed the relevance of this question by intercomparing sorting based on intracellular H-33342 intensity, or based on the ratio of the Hoechst intensity signal to peripheral light scatter signal (essentially, the ratio of 'intensity' to 'volume', or, an estimate of H-33342 'concentration').

Perhaps the most critical assumption made in our sorting procedure is that the various subpopulations of cells in the tumour or spheroid all have the same inherent capacity for uptake of the H-33342, i.e., that the heterogeneity of staining which forms the basis of our ability to separate cells is entirely a function of stain delivery to the cells. There is abundant evidence that functionally different isolated cells show differential uptake of low-concentration Hoechst 33342 (Lalande *et al.*, 1981; Loken, 1980). Unfortunately, we are unable to perform an 'absolute' control experiment to test heterogeneity of uptake within a single tumour: we cannot isolate cells from the different regions of a tumour without using our staining/sorting protocol. We have, however, measured the uptake of additional H-33342 in the separated cell populations, to determine whether under such controlled conditions, differences in cell permeability or other factors influence H-33342 binding.

### Materials and methods

### *Chemicals*

Hoechst 33342 and adriamycin were purchased from Sigma Chemical Co., and stock solutions of each were prepared in sterile distilled water.

### *Spheroids*

Chinese hamster V79 lung fibroblasts were maintained as exponentially growing monolayers in minimal essential medium (MEM) containing 10% foetal bovine serum (FBS) from Gibco. Spheroids were initiated by seeding $2 \times 10^6$ cells into Bellco spinner culture flasks containing 200 ml of MEM plus 5% FBS. Medium was replaced daily after the third day, and spheroids were used for experiments ~10 days after seeding when they were 0.6 to 0.7 mm diam.

For examination of H-33342 loss and transfer, spheroids were exposed for 30 min to 1–25 $\mu$M, and then washed repeatedly by replacing the medium with 200 ml MEM plus 5% FBS every 30 min during the post-incubation period. Spheroids were trypsinized for 8 min at 37°C in 0.25% trypsin, placed in medium, then pipetted vigorously several times to obtain single cells for FACS analysis. For experiments measuring the toxicity of adriamycin, spheroids were incubated for 20 min with 2 $\mu$M H-33342 and then washed and divided into 6 groups. Three were immediately exposed to 5 $\mu$g ml$^{-1}$ adriamycin for 30 mins. The other groups were washed again after 2.5 h and then resuspended in medium containing adriamycin. Single cells obtained from these spheroids were sorted and plated into 100 mm diameter petri dishes containing 10 ml MEM plus 10% FBS. Eight days layer, colonies were stained with malachite green and counted.

### *Mice/tumour*

The SCCVII/St carcinoma originated spontaneously in the abdominal wall of a C3H mouse in the laboratory of Dr H. Suit, Massachussetts General Hospital. Our specimen of the tumour was obtained in 1983 from Dr M. Horseman of Stanford University and has been maintained by inoculation of tumour brei into the gastrocnemius muscle of inbred female C3H/He mice. Tumours required for experimentation were derived by subcutaneous injection of $5 \times 10^5$ viable tumour cells (obtained by enzymic digestion) into the sacral region of the back. Tumours with a mean diameter of 6–8 mm were used in the present study.

H-33342 was dissolved in sterile PBS and injected i.v. into mice (0.25 ml) via the lateral tail vein. Blood levels of H-33342 were measured at various times following i.v. injection of 10 $\mu$g g$^{-1}$ mouse. For each measurement, a 50 $\mu$l blood sample was obtained from the orbital sinus and precipitated in 1 ml of ethanol. After centrifugation, the supernatant was analysed for fluorescence using a Farrand spectrofluorimeter with excitation at 343 nm and emmission at 484 nm. Blood levels in mice were determined by reference to fluorescence intensities obtained using blood samples containing known concentrations of H-33342.

Tumour cell suspensions were prepared by excising the tumour 20 min after i.v. injection of 2 $\mu$g g$^{-1}$ H-33342. The tumour was washed with PBS, and chopped using crossed scalpels. The resulting fragments were then disaggregated by gentle agitation for 30 min at 37°C with an enzyme cocktail of 0.02% trypsin, 0.05% DNAse and 0.05% collagenase. The resulting cell suspension was filtered through polyester mesh (50 $\mu$m pore size), centrifuged, and the cell pellet resuspended in medium for analysis using flow cytometry.

For analysis of tumour cell viability, the soft agar clonogenic assay of Courtenay (1976) was used. The plating efficiency of SCCVII tumour cells was routinely 0.25 to 0.4 when incubated under a 5% oxygen atmosphere.

### *Flow cytometry and sorting*

Cells from tumours and spheroids were analysed and sorted using a Becton Dickinson FACS 440 dual argon laser instrument. H-33342 intensity was measured using excitation at 350–360 nm (40 mW power) with emission monitored with a 449 ± 10 nm band pass filter. In most experiments, the fluorescence intensity of H-33342 stained cells was divided by the peripheral light scatter signal from the 488 nm laser beam (using a 488 ± 10 nm band-pass filter) for each cell to obtain an estimate of cellular 'concentration' of H-33342. In order to recover all of the tumour cells, the sort windows included some normal cells, primarily in the brightest 10–20% of the population. Debris could not be easily distinguished from the dimmest 10% of the population. The fluorescence distributions were generally divided into 10 fractions (sort windows) based on the H-33342 intensity or concetration, with each fraction containing 10% of the population. In addition to the 10 sorted fractions, an 'all-sort' was also collected to measure the average response of the tumour or spheroid cells to treatment.

For experiments using adriamycin, a defined number of cells was sorted into tubes containing 5 ml MEM + 10% FCS which were then poured directly into petri dishes, with two rinses of the same tube. In this way a very accurate measure of the number of cells plated was obtained (Durand, submitted for publication). Adriamycin did not change the H-33342 staining profile in spheroids, and no additional toxicity could be attributed to interactions with H-33342 (Durand, 1981).

### Results

H-33342 concentration in the blood of mice following i.v. administration decreased exponentially with a half-life of 110 sec (Figure 1). The initial blood concentration (~140 μM) was rapidly decreased as H-33342 was bound by tissues and eliminated by the kidney. This rapid removal from the blood suggests that tumours excised 20 min following H-33342 injection will be exposed to negligible free H-33342 during the disaggregation procedure (when the small tumour blood volume is diluted many fold with trypsin), and cells which were distant from the blood supply would therefore remain dimly fluorescent. However, previous studies with V79 cells showed that about half the H-33342 fluorescent was lost from dispersed cells within 2–3 h at 37°C (Durand & Olive, 1982). The possibility therefore existed that H-33342 from brightly-stained cells could transfer to more dimly-stained cells during the staining and disaggregation procedures.

To simulate tumour cord histology in a more

**Figure 1** Blood levels of Hoechst 33342 in C3H mice following i.v. injection of 10 μg g$^{-1}$ mouse. Values of H-33342 less than 0.2 μM could not be detected due to the background fluoresence of the blood.

controlled situation, and where multiple sampling was possible, the 'redistribution' of H-33342 was studied using 0.7 mm diameter Chinese hamster V79 spheroids exposed for 30 min to doses of H-33342 from 1–25 μM (Figure 2). Rather than dissociating spheroids immediately after H-33342 treatment, some were left intact in suspension culture for up to 3 h. Spheroids were given 200 ml fresh medium every 30 min during the post-incubation period to remove any drug lost from the surface (thus simulating the rapid removal of H-33342 from the blood of mice). At specified times, spheroids were trypsinized, and cells were analysed for H-33342 content. As can be seen in Figure 2, about half of the H-33342 was lost by 2 h after treatment, although the remaining stain was lost at a much slower rate, as previously reported for single cells (Durand & Olive, 1982). Since cells stained with 1 μM H-33342 showed the same rate of loss as cells exposed to 25 μM, it seems likely that the extensive media changes during the post-incubation period were adequate to remove most of the H-33342 which was lost into the medium from the surface of the spheroids.

H-33342 lost from the brightly-stained external cells of spheroids may also diffuse further into the spheroid, so that the gradient or differential of drug binding should decrease with time after staining. To measure transfer of H-33342, spheroids were exposed for 30 min to the drug and then left intact

**Figure 2** Loss of Hoechst 33342 from Chinese hamster V79 spheroids. Spheroids were incubated with H-33342 for 30 min at the concentrations indicated. (●) 25 μM; (▲) 10 μM; (△) 5 μM; (○) 1 μM. The mean cellular fluorescence of cells obtained from these spheroids at various times after treatment was determined using flow cytometry.

for up to 3 h. The profile of cell fluorescence through the spheroid was measured by analysing single cells from spheroids for H-33342 content immediately after staining and at subsequent intervals (Figures 3, 4). The external cells of the spheroids were significantly more fluorescent than the internal cells, even 3 h following H-33342 treatment. However, with time after exposure, the external cells lost fluorescence, and the internal cells became more fluorescent. Thus, the gradient of H-33342 binding through the spheroid decreased with time, so that 10% fractions from spheroids dissociated immediately after treatment with 1 μM H-33342 showed a 300-fold range in values for mean cellular fluorescence, but after 3 h, this range was reduced to ~100 (Figure 3a) (i.e., the brightest 10% of the cells were 100 times more fluorescent than the dimmest 10%). Similar results were obtained for 25 μM H-33342 with a decrease in heterogeneity of binding from ~200 to 35.

Figure 4 shows additional data for cells from individual sort windows, again indicating that internal cells of spheroids became more fluorescent with time after H-33342 treatment, even though the average cellular response was loss of fluorescence (dotted line in Figure 4). In fact, for spheroids exposed to 1 μM H-33342, 70% of the cells of the spheroid showed an increase in fluorescence intensity although the mean cellular fluorescence of all cells decreased by 50% after 3 h. However, while H-33342 redistribution occurred, it is apparent that a large gradient of binding through the spheroid remained even 3 h following treatment.

To evaluate whether this gradient remained adequate to distinguish internal from external cells of spheroids, spheroids first exposed to H-33342 for 20 min were then treated immediately with 5 $\mu g\,ml^{-1}$ adriamycin, or were left for 2.5 h before incubation with adriamycin. Adriamycin was chosen for two reasons. First, previous studies using H-33342 to sort cells from spheroids showed that adriamycin penetrated poorly into spheroids so that a steep gradient of cell killing was observed (Durand, 1981). Such a gradient is convenient for determining whether the H-33342 gradient was adequate for cell separation (i.e., if all cells of a spheroid responded identically to adriamycin, then the resolving power of H-33342 could not be tested). The second reason for using adriamycin is that work by Preisler (1978) suggested an interaction between H-33342 and adriamycin which we wanted to investigate at the lower stain concentrations used in our system. Three separate populations of spheroids were indepedently exposed to adriamycin at both times, in order to evaluate the reproducibility of the technique. Spheroids were then sorted on the basis of H-33342 concentration into 10 sort windows which were analysed for adriamycin-induced cell killing. As shown in Figure 5, reproducibility was excellent and the *average* response of spheroids exposed to adriamycin immediately or 2.5 h after H-33342 treatment was not significantly different. Therefore, average cell survival was independent of when the spheroids saw H-33342, showing that H-33342 is non-toxic and suggesting that it does not interact with adriamycin. Previous results comparing the toxicity of adriamycin with or without H-33342 in spheroids support this conclusion (Durand, 1982). While both panels showed greater killing of external cells by adriamycin, there was greater resolution (as defined by greater differential survival) in spheroids treated immediately after H-33342 exposure (the decrease in survival in window 10 is the result of inclusion of cell debris). More toxicity was observed in the brightly stained spheroid cells sorted immediately after H-33342 treatment than 2.5 h later, suggesting some redistribution of H-33342 in these cells with time after H-33342 treatment. However, since tumour cells are routinely sorted within 1 h of excision, redistribution of H-33342 is not likely to interfere significantly with resolution of cell populations on the basis of distance from the blood supply.

**Figure 3** Transfer of Hoechst 33342 between cells of spheroids. Spheroids were exposed to (a) 1 μM or (b) 25 μM H-33342 for 30 min and then either disaggregated immediately (0 h; ●) or 3 h after treatment (▲). Single cells from these spheroids were analysed for fluorescence using flow cytometry. Each sort window represents 10% of the population, with the most fluorescent cells being the external cells of the spheroid.

**Figure 4** Transfer of Hoechst between cells of spheroids. Spheroids were exposed to (a) 1 μM or (b) 25 μM H-33342 for 30 min, then disaggregated at various times after treatment. Cells were sorted, on the basis of fluorescence, into windows representing 10% of the population (see **Figure 3**). These windows were then analysed for fluorescence intensity. Selected windows are designated on the left hand side of each panel. The dotted line represents the average response of all of the cells ('all-sort').

**Figure 5** Response of spheroids to adriamycin immediately and 2.5 h after exposure to Hoechst 33342. Spheroids were exposed to 2 μM H-33342 for 20 min, washed and incubated either immediately with 5 μg ml$^{-1}$ adriamycin for 30 min (a) or 2.5 h later (b). The three different symbols represent 3 different populations exposed independently to adriamycin. Symbols at sort window '0' represent the average response of all of the cells of the spheroids. Cell debris is included in window 10.

With cells of spheroids, binding of H-33342 appears exclusively dependent on cell position within the spheroid. However, in more heterogeneous cell systems such as tumours, other factors such as cell permeability (Loken, 1980; Lalande *et al.*, 1981) might influence binding of H-33342 so that H-33342 content or even concentration (ratio of intensity to cell size) may not accurately indicate the position of a cell relative to the blood supply. If binding of H-33342 to subpopulations of tumours is dependent on factors other than cell size, then the rate of binding should differ in tumour cell subpopulations sorted on the basis of H-33342 content, and exposed *in vitro* to additional H-33342. Therefore, an SCCVII tumour was exposed to H-33342 by i.v. injection of 10 μg g$^{-1}$. The tumour was excised and single cells were analysed for H-33342 content as shown in Figure 6a. Tumour cells were then sorted into 4 equal populations on the basis of H-33342, and re-exposed to additional H-33342. Subsequent binding rates varied ~2-fold with cells from poorly vascularized areas binding at about half the rate as cells next to the vasculature, probably due to differences in cell volume. Therefore, uptake of H-33342 *in situ*, at least in the SCCVII tumour, appears to depend primarily on cell location relative to the blood supply.

Finally, to address the possible toxicity of H-33342, SCCVII tumour cells were exposed *in vitro* for 2 h to H-33342, and then clonogenicity and mean cell fluoresence were measured. Concentrations of H-33342 greater than 30 μM, corresponding to a mean cell fluorescence ~500 times the background, were required to produce significant cell killing (Figure 7). Since the brightest 10% of tumour cells exposed *in situ* are only ~10 times more fluorescent than background (Figure 6a), it is apparent that we are well below concentrations of H-33342 necessary for toxicity to these cells.

### Discussion

H-33342 appears to be a very useful agent for viable cell selection and sorting. It is non-toxic at the concentrations required for tumour cell sorting, penetrates slowly into poorly vascularized tissue, and is also lost slowly from these tissues. Redistribution does occur but is minimal over the 30–60 min required for tumour disaggregation and does not significantly affect the resolution of cell position through the tumour cord.

Previous studies have indicated that not all cells bind H-33342 to the same extent, even those cells containing the same amount of DNA (Loken, 1980; Lalande *et al.*, 1981). Differences in permeability of cells to H-33342 by different CHO cell lines resulted in a 7-fold difference in cellular fluor-

**Figure 6** Uptake of Hoechst 33342 by SCCVII tumour cells. A C3H mouse bearing a 350 mg SCCVII tumour was injected i.v. with $2 \mu g g^{-1}$ H-33342 20 min before removing the tumour. The tumour cells were analysed for fluorescence intensity (a) as in **Figure 3.** Cells were divided according to fluorescence intensity into 4 sort windows each representing 25% of the population. $2 \times 10^5$ cells were sorted and reincubated with 0.5 $\mu$M H-33342 for various times, when samples were removed for analysis of mean cell fluorescence (b). (○) brightest 25%; (▲) 50–75%; (△) 25–50%; (●) dimmest 25%.

**Figure 7** Toxicity of Hoechst 33342 to SCCVII tumour cells *in vitro*. Cells were incubated for 2 h with H-33342 at 37°C, and then analysed for clonogenic potential (a) and for fluorescence intensity (b).

escence. However, from the data shown in Figure 6, it seems unlikely that differences between cells change the rate of H-33342 uptake by more than a factor of 2, at least in the SCCVII tumour. Since the gradient of drug binding represents a 20–100 fold difference across the tumour, we have not been concerned with small variations that might occur between heterogeneous cells exposed to the same concentration of H-33342. However, this conclusion is based on the assumption that uptake by single cells obtained from tumours is the same as uptake by these cells *in situ*. We are unable to verify whether this is indeed true, just as we cannot determine absolutely whether the presence of small amounts of H-33342 in cells interferes with the toxicity of drugs and radiation (since the sort cannot be performed without H-33342). Indirect evidence can be obtained by comparing results with different doses of H-33342, and assuming that interactions between H-33342 and drugs should increase as the concentration of H-33342 increases. Preisler *et al.* (1978) have suggested that H-33342 and adriamycin may bind to DNA by similar mechanisms, and H-33342 treatment might therefore interact with adriamycin. However, in their studies, adriamycin was given before H-33342 (20 $\mu$M) and appeared to inhibit H-33342 binding to human leukaemic cells. At the dose of H-33342 (2 $\mu$M) and time of treatment (20 min) used in these studies, we observed no change in survival of cells treated with adriamycin immediately after H-33342 exposure or 2.5 h later (when the amount of H-33342 remaining in the cell had decreased by 50%).

The possibility of using H-33342 to measure tumour perfusion in a quantitative way is also being explored. Such studies can be performed by measuring mean cellular fluorescence using flow cytometry with tumour cells stained *in situ*, but could also be performed by analysing frozen sections of tumours using microspectrofluorimetry. In larger tumours, the use of a complementary stain may be necessary to accurately resolve the position of tumour cells distant from the blood supply. Unlike spheroids, where the external cells directly exposed to H-33342 constitute 10% or more of the spheroid, the proportion of tumour cells which are directly exposed to H-33342 is considerably smaller (i.e., the difference between inward flow with spherical symmetry and outward flow with cylindrical symmetry: see Boag, 1969). This point is illustrated in Figures 3 and 6 where spheroids and SCCVII tumours were incubated with H-33342. In Figure 3, spheroids show a 300-fold difference between the dimmest and brightest 10% of cells, while in Figure 6, there is only a 20-fold difference. Similarly, the mean cellular fluorescence is considerably smaller for tumour cells than for spheroid cells. While transfer could contribute to the decrease in the H-33342 gradient in tumours, it seems more likely that differences in diffusion patterns explain these discrepancies. The majority of cells in a large tumour with poor perfusion are exposed to a much smaller concentration of H-33342 which may not provide an adequate gradient for effectively sorting cells distant from the tumour vasculature. To overcome this problem, other fluorescent stains (excited by visible light) are being examined which will preferentially stain cells distant from the blood supply. Potential stains include fluorescent probes for hypoxia (Olive & Durand, 1983) or fluorescent drugs such as fluorescein diacetate which appear to accumulate in cells at low pH (Chaplin, unpublished results). When combined with H-33342, such complementary stains should greatly enhance the gradient for cell sorting.

The authors acknowledge the expert technical assistance of Denise McDougal, Nancy Arnold and Doug Aoki. This research was supported by grant numbers CA37879 and CA37775 awarded by the National Cancer Institute, DHHS, and by the National Cancer Institute of Canada.

### References

ARNDT-JOVIN, D.J. & JOVIN, T.M. (1977). Analysis and sorting of living cells according to deoxyribonucleic acid content. *J. Histochem. Cytochem.*, **25**, 585.

BOAG, J.W. (1969). Oxygen diffusion and oxygen depletion problems in radiobiology. *Curr. Top. Radiat. Res.*, **5**, 141.

CHAPLIN, D.J., DURAND, R.E. & OLIVE, P.L. (1985). Cell selection from a murine tumour using the fluorescent probe Hoechst 33342. *Br. J. Cancer*, **51**, 569.

COURTENAY, V.D. (1976). A soft agar colony assay for Lewis lung tumour and B16 melanoma taken directly from the mouse. *Br. J. Cancer*, **34**, 39.

DURAND, R.E. (1981). Flow cytometry studies of intracellular Adriamycin in multicell spheroids *in vitro*. *Cancer Res.*, **41**, 3495.

DURAND, R.E. (1982). Use of Hoechst 33342 for cell selection from multicell systems. *J. Histochem. Cytochem.*, **30**, 117.

DURAND, R.E. & OLIVE, P.L. (1981). Flow cytometry studies of intracellular adriamycin in single cells *in vitro*. *Cancer Res.*, **41**, 3489.

DURAND, R.E. & OLIVE, P.L. (1982). Cytotoxicity, mutagenicity and DNA damage by Hoechst 33342. *J. Histochem. Cytochem.*, **30**, 111.

LALANDE, M.E., LING, V. & MILLER, R.G. (1981). Hoechst 33342 dye uptake as a probe of membrane permeability changes in mammalian cells. *Proc. Nat. Acad. Sci.*, **78**, 363.

LÖKEN, M.R. (1980). Separation of viable T and B lymphocytes using a cytochemical stain, Hoechst 33342. *J. Histochem. Chtochem.*, **28**, 36.

OLIVE, P.L. (1985). Fluorescent probes for cellular hypoxia: lack of transfer of fluorescence between cells *in vitro*. *Int. J. Radiat. Oncol. Biol. Phys.*, in press.

OLIVE, P.L. & DURAND, R.E. (1983). Fluorescent nitroheterocycles for identifying hypoxic cells. *Cancer Res.*, **43**, 3276.

PALLAVICINI, M.G., LALANDE, M.E., MILLER, R.G. & HILL, R.P. (1979). Cell cycle distribution of chronically hypoxic cells and determination of the clonogenic potential of cells accumulated in $G_2+M$ Phases after irradiation of a solid tumor *in vivo*. *Cancer Res.*, **39**, 1891.

PRIESLER, H.D. (1978). Alteration of binding of the supravital dye Hoechst 33342 to human leukemic cells by adriamycin. *Cancer Treatment Rep.*, **62**, 1393.

REINHOLD, H.S. & VISSER, J.W.M. (1983). *In vivo* fluorescence of endothelial cell nuclei stained with the dye bisbenzamide Hoeschst 33342. *Int. J. Microcirc: Clin. Exp.*, **2**, 143.

SMITH, P.J. & ANDERSON, C.O. (1984). Modification of the radiation sensitivity of human tumour cells by a bis-benzimidazole derivative. *Int. J. Radiat. Biol.*, **46**, 331.

SZABO, G., KISS, A. & DAMJANOVICH, S. (1981). Flow cytometric analysis of the uptake of Hoechst 33342 dye by human lymphocytes. *Cytometry*, **2**, 20.